# Common low-penetrance risk variants associated with breast cancer in Polish women

**DOI:** 10.1186/1471-2407-13-510

**Published:** 2013-10-30

**Authors:** Joanna K Ledwoń, Ewa E Hennig, Natalia Maryan, Krzysztof Goryca, Dorota Nowakowska, Anna Niwińska, Jerzy Ostrowski

**Affiliations:** 1 Department of Gastroenterology and Hepatology, Medical Center for Postgraduate Education, Warsaw, Poland; 2 Department of Genetics, Maria Sklodowska-Curie Memorial Cancer Center and Institute of Oncology, Warsaw, Poland; 3 Genetic Counseling Unit, Maria Sklodowska-Curie Memorial Cancer Center and Institute of Oncology, Warsaw, Poland; 4 Department of Breast Cancer and Reconstructive Surgery, Maria Sklodowska-Curie Memorial Cancer Center and Institute of Oncology, Warsaw, Poland

**Keywords:** Breast cancer, Cancer susceptibility, Single nucleotide polymorphism, Genetic associations

## Abstract

**Background:**

Breast cancer is the most common type of cancer and the second leading cause of cancer-death among women in Poland. The known high-risk mutations account for 25% of familial aggregation cases and 5% of total breast cancer predisposition. Genome-wide association studies have identified a number of common low-penetrance genetic variants, but their contribution to disease risk differs between populations.

**Methods:**

To verify selected associations with breast cancer susceptibility among Polish women, the replication study was performed, included 1424 women with breast cancer and 1788 healthy persons. Sixteen single-nucleotide polymorphisms (SNPs) were analyzed using TaqMan SNP Genotyping Assays. Allele frequency differences were tested using chi^2^-test implemented in PLINK v1.07 and Cochran-Armitage trend test was performed using R software.

**Results:**

Significant differences (Bonferroni corrected *p*-value_cor_ ≤ 0.0197) in the frequency of alleles distribution between all cancer and control subjects were observed for four (rs2736098, rs13281615, rs1219648, rs2981582) out of 16 SNPs. The same result was obtained for group of patients without high-risk *BRCA1/2* mutations. The rs1219648 (*p*-value_cor_ ≤ 6.73E-03) and rs2981582 (*p*-value_cor_ ≤ 6.48E-03) SNPs showed significant association with both familial and sporadic cancers. Additionally, rs2736098 (*p*-value_cor_ ≤ 0.0234) was associated with only sporadic cancers; also in group without carriers of high-risk mutation. All these associations revealed their significance also in Cochran-Armitage trend test. Opposite to other SNPs, rs2736098 was associated with a decreased risk of breast cancer.

**Conclusion:**

The association of four known susceptibility SNPs, representing three individual *loci*, with breast cancer risk in Polish women was confirmed. One of them (rs2736098) seems to be specific for the Polish population. Due to the population differences in allele frequencies, identification of general genetic risk factors requires sets of association studies conducted on different populations.

## Background

Breast cancer is the most commonly occurring cancer among women worldwide [1]. In Poland it accounts for over 20% of all malignant tumors and is the second most frequent cause of cancer-related death [2]. Although the majority of breast cancer cases are sporadic, a noticeable portion results from highly penetrating inherited mutation in susceptibility genes and family history remains the best predictor of their individual risk [3]. Among known predisposition genes, deleterious mutations in *BRCA1* and *BRCA2* confer the strongest effect on disease susceptibility and are associated with a lifetime risk of breast cancer of up to 85% for such mutation carriers [4,5].

Even though the impact of high-risk gene mutations is noticeable, they account for only about 25% of the familial risk and less than 5% of total breast cancer predisposition, as their frequencies in general population are very low [6]. It is suggested that remaining risk may result from a combination of multiple common variants, each conferring a small effect on breast cancer risk, with odds ratio (OR) usually between 1.2 and 1.5 [7,8]. According to the polygenic model, a large number of low-penetrance variants may have cumulative effect on both the overall risk of disease [9] and an early disease onset [10,11].

A number of common single-nucleotide polymorphisms (SNPs) associated with slightly modified risk of different cancers have been identified through genome-wide association studies (GWAS). By far, at least 22 GWAS were conducted for breast cancer on different populations revealing over 36 susceptibility *loci*[12]. Fifteen of them were consistently confirmed in other GWAS or large replication studies and meta-analyses [11,13-24].

Conducting analyses on different populations increases the chance for generalization of conclusions and identification of causal variants [25]. For its apparently high level of genetic homogeneity [26,27], the Polish population seems to be relevant for determining risk variants with relatively small, although significant, effect on cancer prevalence. In this study we focus on verification of selected associations with breast cancer risk among Polish women. Eleven SNPs were chosen for replication as commonly reported in different studies. Additional five variants were selected for evaluation based on data provided by Genetic Counseling of Cancer Center-Institute of Oncology in Warsaw as frequently observed in patients treated in Cancer Center. To our knowledge, by now only two of these SNPs were investigated for association with breast cancer susceptibility in Poland.

## Methods

### Studied population

The study was conducted at the Cancer Center-Institute of Oncology in Warsaw and blood samples were collected between 2003–2010. In total, 3212 women were included: 1424 with newly-diagnosed breast cancer (992 of less than 50 years of age at diagnosis) and 1788 healthy individuals. The personal and familial cancer history was acquired by comprehensive interviews for all patients. Cases representing families with at least one breast or ovarian cancer diagnosis in a first- or second-degree relative were considered as familial breast cancer. Patients with less than 50 years of age at the moment of diagnosis were considered as early-onset cases. The detailed study groups statistics are presented in Table 1.

**Table 1:** Group statistics of study cohorts

	High risk mutation carriers^*^	**No *BRCA1/2 *** **mutation carriers**	**Total**	**Median age**
Familial BCa (with family history)	168	617	785	43 (29–65)
Sporadic BCa (without family history)	75	564	639	45 (17–62)
All BCa cases	243	1181	1424	44 (17–65)
Controls	-	-	1788	58 (26–79)

^*^ Women with any of *BRCA1* mutations presented in Table 2. None of patients carried any of selected *BRCA2* mutations.

All patients were tested for selected pathogenic mutations in *BRCA1* and *BRCA2* chosen as the most frequently occurred among Polish women with breast cancer [26,28]. For *BRCA1*, the whole sequence of exons 2, 5 and 20, and a part of exon 11 (nucleotides 2893 to 3502 from the beginning of this exon) were analyzed. The sequences of primers used for amplification of relevant fragments are listed in Additional file 1: Table S1. All identified mutations are presented in Table 2; women with at least one of these mutations were further considered as high-risk mutation carriers. For *BRCA2*, selected 11 mutations (G1408T, 5467insT, 6174delT, 6192delAT, 6675delTA, 8138del5, 9152delT, 9182-2A>G, 9326insA, C9610T, 9631delC) were directly sequenced and none of included patients carried any of these mutations. Healthy women were recruited primarily from the National Colorectal Cancer Screening Program, which enrolls healthy persons from the general population aged 50 years and older. All women exhibited no known history of cancer and normal results of mammography and screening colonoscopy. All patients and control subjects were Polish Caucasians recruited from two urban populations, Warsaw and Szczecin. The study was approved by the local ethics committee (Medical Center for Postgraduate Education and Cancer Center-Institute of Oncology, Warsaw, Poland) and all participants provided written informed consent. The study protocol conforms to the ethical guidelines of the 1975 Declaration of Helsinki.

**Table 2:** **Mutations detected in the selected regions of *BRCA1 *** **gene among study participants**

**Exon 2**	**Exon 5**	Exon 11 – part^*^	**Exon 20**
185delAG	T300G	3819del5	G5332A
		3875del4	C5370T
		4153delA	5382insC
		4160delAG	
		4184del4	

^*^Nucleotides 2893 to 3502 from the beginning of the exon 11 of *BRCA1* gene.

### SNP selection

Sixteen SNPs were selected for replication among Polish women (Table 3); 11 SNPs were chosen from the literature as consistently shown to be associated with breast cancer risk in various studies (8 SNPs from GWAS and 3 from candidate gene studies) [8,29-35]. Additional five SNPs were selected based on data provided by Genetic Counseling of Cancer Center-Institute of Oncology in Warsaw, Poland, as relatively frequent among coming forward patients. All five are missense variants in three genes: *BRCA2* (3 SNPs), *PALB2* and *CDKN2A.* One of these SNPs (rs1799944 in *BRCA2*) was previously reported to be associated with breast cancer in Cyprus [36]. For rs3731249 in *CDKN2A,* contribution to early onset breast cancer in Poland was suggested [37]. Remaining four SNPs have not, so far, been studied for association with breast cancer risk among Polish women.

**Table 3:** SNPs selected for analysis

**NCBI SNP Reference**	**Cytogenetic Band**	**Gene**^**a**^	**Reference**
rs17468277	2q33.1	*ALS2CR12* (synonymous) (*CASP8*)^b^	[35]
rs13387042	2q35	intergenic	[32,33]
rs889312	5q11.2	*MAP3K1* (upstream)	[8,33]
rs10941679	5p12	intergenic	[8,34]
rs2736098	5p15.33	*TERT* (synonymous)	[31]
rs13281615	8q24.21	intergenic	[8,33]
rs3731249	9p21.3	*CDKN2A* (missense; A148T)	[37], CO-I^c^
rs1219648	10q26	*FGFR2* (intron)	[30]
rs2981582	10q26	*FGFR2* (intron)	[8,33]
rs3817198	11p15.5	*LSP1* (intron)	[8]
rs766173	13q13.1	*BRCA2* (missense; N289H)	CO-I
rs1799944	13q13.1	*BRCA2* (missense; N991D)	[36], CO-I
rs28897710	13q13.1	*BRCA2* (missense; T598A)	CO-I
rs3803662	16q12.1	*TOX3/LOC643714* (between)	[8,32,33]
rs243865	16q13-q21	*MMP2* (promoter)	[29]
rs152451	16p12.2	*PALB2* (missense; Q559R)	CO-I

^a/^NCBI ID of genes localized in proximity to the SNPs of interest (source: HapMap).

^b/^SNP rs17468277 is in strong LD (*r*^*2*^ = 1) with rs1045485 in *CASP8* (D302H)*.*

^c/^SNP selected based on the date provided by Genetic Counseling of Cancer Center and Institute of Oncology (CO-I) in Warsaw.

### Genotyping

Genomic DNA was extracted from whole blood treated with EDTA using the QIAamp DNA mini Kit (Qiagen, Gernamy), following the manufacturer’s protocol. DNA samples quantity and quality were evaluated using a NanoDrop 1000 spectrophotometer (Thermo Fisher Scientific Inc., USA). The samples which passed quality control were adjusted to a final concentration of 50 ng/μl in Tris–EDTA buffer (pH = 8), with concentrations of Tris and EDTA not exceeding limits of 10 and 0.1 mM, respectively. Individual genotyping was performed using TaqMan SNP Genotyping Assays (Life Technologies, USA), SensiMix™ II Probe Kit (Bioline Ltd, United Kingdom) and a 7900HT Real-Time PCR system (Life Technologies, USA) in 384-well format.

### Statistical analyses

Quality control of TaqMan genotyping results included: thresholds for maximum individual missingness for each of the SNPs < 0.05, maximum genotype missingness for each of the individuals < 0.05 and the Hardy-Weinberg disequilibrium < 0.001 for the control group. Associations were examined using allelic chi^2^-test implemented in PLINK v1.07 software (http://pngu.mgh.harvard.edu/purcell/plink/). Furthermore, the Cochran-Armitage trend test was performed using R software (http://www.r-project.org/), "coin" library. OR 95% confidence interval (CI) was estimated by normal approximation implemented in "epitools" package. The Bonferroni correction was used for multiple comparisons and *p-*value_cor_ < 0.05 was considered significant. The study sample size calculations were conducted with tools supported in "pwr" library, assuming equal sizes of groups and baseline allele frequency equal to frequency observed in control group. Calculations were performed for power equal 0.8 and significance threshold equal 0.05. Results of sample size calculations are presented in Additional file 2: Table S2.

## Results

The vast majority of 1424 women with breast cancer included in this study were non high-risk *BRCA1* or *BRCA2* mutation carriers: only 243 (17.1%) patients had one of *BRCA1* mutations indicated in Table 2 and none of them had any of 11 genotyped mutations in *BRCA2*. The number of patients with family cancer history was similar to the number of sporadic tumor cases (785 *vs.* 639), as shown in Table 1. The median age at diagnosis was 44, ranging from 17 to 85.

The differences (*p*-value ≤ 0.0314) were observed for seven out of 16 SNPs when allele frequencies between all cases and control subjects were assessed with the chi^2^-test and four of them (rs2736098, rs13281615, rs1219648, rs2981582) remained significantly associated after multiple testing adjustment (*p*-value_cor_ ≤ 0.0197) (Table 4 and Additional file 3: Table S3 for all results). The same four SNPs show significant association (*p*-value_cor_ ≤ 0.0116) with breast cancer susceptibility in group of patients tested negative for the high-risk mutations. The strongest association was observed for rs1219648 and rs2981582 (*p-*value_cor_ of 1.62E-05 and 1.46E-05, respectively). Both are located in intron 2 of *FGFR2* encoding the fibroblast growth factor receptor 2 protein. The minor allele of rs2736098 located in *TERT* gene was associated (*p*-value_cor_ of 9.30E-04) with a decreased risk of breast cancer. Fourth SNP (rs13281615) was located in 8q24 locus, known as multicancer susceptibility region [38]. None of the 16 SNPs showed association after Bonferroni correction in the group of *BRCA1* mutation carriers.

**Table 4:** The significant SNP associations with breast cancer considering allelic and Cochran-Armitage trend tests

**dbSNP ID**^**a**^	**Region**	**Gene**^**b**^	**MA**	**G1 vs G2**	**OR (95% CI)**	**Allelic**		**Cochran-Armitage**	
						***p*-value**	***p*-value**_**cor**_	***p*-value**	***p*-value**_**cor**_
rs10941679	5p12		0.24	C vs N	1.14 (1.01-1.28)	2.97E-02	4.75E-01	2.71E-02	4.34E-01
				C noMut vs N	1.15 (1.02-1.30)	2.29E-02	3.66E-01	2.04E-02	3.27E-01
				S vs N	1.17 (1.01-1.35)	4.25E-02	6.80E-01	3.94E-02	6.31E-01
				S noMut vs N	1.20 (1.03-1.40)	2.06E-02	3.29E-01	1.87E-02	2.99E-01
rs2736098	5p15.33	*TERT*	0.36	C vs N	0.77 (0.68-0.88)	5.81E-05	**9.30E-04**	5.51E-05	**8.82E-04**
				C noMut vs N	0.78 (0.69-0.89)	2.37E-04	**3.78E-03**	2.25E-04	**3.60E-03**
				C with Mut vs N	0.74 (0.57-0.94)	1.70E-02	2.73E-01	1.55E-02	2.48E-01
				F vs N	0.81 (0.69-0.93)	4.68E-03	7.49E-02	4.56E-03	7.30E-02
				F noMut vs N	0.80 (0.68-0.94)	8.63E-03	1.38E-01	8.25E-03	1.32E-01
				S vs N	0.74 (0.63-0.87)	2.61E-04	**4.17E-03**	2.38E-04	**3.80E-03**
				S noMut vs N	0.76 (0.64-0.90)	1.46E-03	**2.34E-02**	1.36E-03	**2.18E-02**
rs13281615	8q24.21		0.45	C vs N	1.19 (1.07-1.32)	1.23E-03	**1.97E-02**	1.17E-03	**1.88E-02**
				C noMut vs N	1.21 (1.08-1.35)	7.23E-04	**1.16E-02**	6.77E-04	**1.08E-02**
				F vs N	1.20 (1.06-1.35)	5.19E-03	8.31E-02	5.24E-03	8.39E-02
				F noMut vs N	1.22 (1.06-1.39)	4.60E-03	7.36E-02	4.53E-03	7.25E-02
				S vs N	1.18 (1.03-1.35)	1.61E-02	2.57E-01	1.42E-02	2.27E-01
				S noMut vs N	1.20 (1.04-1.38)	1.09E-02	1.74E-01	9.61E-03	1.54E-01
rs1219648	10q26	*FGFR2*	0.41	C vs N	1.30 (1.17-1.45)	1.01E-06	**1.62E-05**	1.13E-06	**1.81E-05**
				C noMut vs N	1.36 (1.22-1.52)	7.20E-08	**1.15E-06**	7.95E-08	**1.27E-06**
				F vs N	1.26 (1.11-1.43)	4.21E-04	**6.73E-03**	3.76E-04	**6.01E-03**
				F noMut vs N	1.33 (1.16-1.53)	4.02E-05	**6.43E-04**	3.24E-05	**5.18E-04**
				S vs N	1.36 (1.19-1.56)	7.32E-06	**1.17E-04**	8.53E-06	**1.36E-04**
				S noMut vs N	1.39 (1.20-1.59)	5.58E-06	**8.92E-05**	6.76E-06	**1.08E-04**
rs2981582	10q26	*FGFR2*	0.41	C vs N	1.31 (1.17-1.45)	9.10E-07	**1.46E-05**	1.17E-06	**1.88E-05**
				C noMut vs N	1.35 (1.21-1.51)	1.20E-07	**1.91E-06**	1.54E-07	**2.46E-06**
				F vs N	1.26 (1.11-1.43)	4.05E-04	**6.48E-03**	4.09E-04	**6.54E-03**
				F noMut vs N	1.32 (1.15-1.51)	6.49E-05	**1.04E-03**	6.11E-05	**9.77E-04**
				S vs N	1.37 (1.19-1.56)	5.70E-06	**9.12E-05**	7.67E-06	**1.23E-04**
				S noMut vs N	1.38 (1.20-1.59)	5.69E-06	**9.11E-05**	7.97E-06	**1.27E-04**
rs3817198	11p15.5	*LSP1*	0.34	F vs N	1.16 (1.02-1.32)	2.45E-02	3.92E-01	2.36E-02	3.78E-01
				F noMut vs N	1.16 (1.00-1.33)	4.46E-02	7.14E-01	4.34E-02	6.94E-01
rs3803662	16q12.1	*TOX3*	0.30	C vs N	1.13 (1.01-1.27)	3.14E-02	5.02E-01	3.22E-02	5.15E-01
				C noMut vs N	1.16 (1.03-1.31)	1.30E-02	2.08E-01	1.35E-02	2.16E-01
				F noMut vs N	1.16 (1.00-1.34)	4.45E-02	7.11E-01	4.53E-02	7.25E-01
				S noMut vs N	1.16 (1.00-1.35)	4.75E-02	7.61E-01	4.82E-02	7.71E-01

Bold denotes significant association after multiple testing adjustment (*p-*value_*cor*_ < 0.05). G1 vs. G2; compared groups of cases and controls, respectively, MA; minor allele (+) strand frequency, OR; odds ratio, CI; confidence interval, N; control, C; cancer (all cases), F; familial cancer, S; sporadic cancer, noMut; non-mutation carriers.

^a/^SNP identifier based on NCBI SNP database;

^b/^NCBI ID of genes localized in proximity to the SNPs of interest (source: HapMap).

SNPs rs2981582 and rs766173 are in the same linkage disequilibrium (LD) blocks with rs1219648 (*r*^*2*^ = 0,967) and rs1799944 (*r*^*2*^ = 1), respectively [39]. Consistent with expectations, both pairs indicate similar associations.

To further explore associations with breast cancer, we performed analyses separately in groups of familial and sporadic cases, with additional stratification based on mutations in high-risk genes. Two SNPs in *FGFR2* show significant association with both familial and sporadic cases (*p-*value_cor_ ≤ 6.73E-03) (Table 4). Additionally, rs2736098 (*TERT*) was associated with sporadic cancers only (*p-*value_cor_ ≤ 0.0234). Results for both types of breast cancer did not change when carriers of high-risk mutation were excluded. All significant associations obtained in the chi^2^-test were confirmed by the Cochran-Armitage trend test analysis (Table 4).

## Discussion

Several association studies support the polygenic inheritance model of breast cancer, showing increasing risk of disease when many predisposition variants of low effect size were combined [11,40]. However, strong bias of the association results, by highly penetrant genetic determinants, such as deleterious mutation in *BRCA1* or *BRCA2* gene, should be taken into account. Also, significant modification of breast cancer risk in *BRCA1/2* mutation carriers was observed in association with selected low-penetrant risk alleles [41].

In this replication study, association of selected susceptibility SNPs with both familial and sporadic breast cancers was analyzed. Among studied patients, at least one from nine different *BRCA1* mutations was shown in over 11% of sporadic cases and 21% of familial cancers, which is in agreement with previous findings for women in Poland [28].

From 16 susceptibility variants selected for analysis, four SNPs, representing three different *loci*, significantly associated (*p*-value_cor_ < 0.05) with breast cancer risk, both in group of all cases as in sporadic and familial cancer subgroups, and after exclusion of *BRCA1* mutation carriers (Table 4). Two SNPs (rs1219648 and rs2981582) lie within intron 2 of *FGFR2* gene, encoding a receptor tyrosine kinase which participates in activation of signaling pathways engaged in tumor induction and progression [42] and mediates breast cancer cell proliferation through D-type cyclins [43]. Amplification or overexpression of *FGFR2* was observed in 5-10% of breast tumors [44] and breast cancer cell lines [45].

The association of *FGFR2* variants with breast cancer risk was reported in several studies and is very well documented, with the strongest association observed for rs2981582 [8,30]. Minor allele of rs2981582 was found to correlate with positive family history of breast cancer [46-48] and early-onset of non-familial breast cancer [47]. Data provided by The Consortium of Investigators of Modifiers of BRCA1/2 (CIMBA) indicated that the *FGFR2* locus associated with breast cancer in *BRCA2* mutation carriers but not in *BRCA1* mutation carriers [49,50]. This finding may reflect the differences in the distribution of tumor subtype. Rs2981582 is consistently most strongly associated with the estrogen receptor (ER)-positive/low grade tumors [23], which are more typical for *BRCA2* mutation carriers and general population not selected for carrier status [51]. Consistently, our findings indicated higher OR and stronger association of both *FGFR2* variants in groups of patients without *BRCA1* mutations (Table 4). Possible correlation of *FGFR2* risk alleles with gene expression was suggested, as intron 2 contains several putative transcription-factor binding sites [52]. However, relationship of risk allele rs2981582 with increased expression of *FGFR2* has not been clarified yet [52,53].

SNP rs13281615 is located in a ‘gene desert’ on chromosome 8q24, where five independent cancer susceptibility *loci* were identified so far [54]. One *loci*, termed ‘region 2’ (stretched from 128.35-128.51 Mb), is specific for breast cancer only and tagged by rs13281615 [38]. Gene desert at 8q24 is located few hundred kilo bases telomeric to the proto-oncogene *Myc*. As multiple enhancer elements were identified in this region, it was suggested that they can regulate the transcription of *Myc*. One such element was shown to physically interact with *Myc* promoter via Tcf-4 transcription factor binding and this interaction affected c-*Myc* expression in an allele specific manner [55]. Overexpression of c-*Myc* was observed in breast cancer tissue [56]; its reduction inhibited breast tumor cells growth [57]. In agreement with our findings, SNP rs13281615 was associated with increased risk of breast cancer among people at higher risk (who have positive family cancer history or *BRCA1/BRCA2* mutation) [17,48]. It was also shown that the association of rs13281615 was stronger for ER-positive disease, with no evidence of an association for ER-negative disease [58], although, no association with either *BRCA1* or *BRCA2* carriers was observed [19].

In recent years, the association of rs2736098 (5p15) with cancer risk at different locations was reported, especially for lung and bladder cancers [59-61]. For breast cancer, the reported findings have been controversial [31,60,62]. Haiman et al. [62] observed positive association of 5p15 locus with increased risk of breast cancer. In turn, Savage et al. [31] suggested protective effect of three correlated SNPs in this region, including rs2736098, among Polish women with positive family history. Similarly, in our study, rs2736098 minor allele was associated with reduced overall and sporadic breast cancer risk. For familial cancers, association was also observed, although not statistically significant after Bonferroni adjustment.

Rs2736098 is located in coding sequence of *TERT* gene, therefore it has been considered as a putative cancer susceptibility gene. *TERT* encodes the catalytic subunit of telomerase, which is crucial in cellular proliferation because counteracts telomere-dependent replicative aging [63]. In many types of cancer, *TERT* shows a high-level of expression, which possibly induces excessive cell growth and carcinogenesis [64]. Although rs2736098 is a synonymous polymorphism, it has been shown to be correlated with telomere length, however not with *TERT* expression [59]. On the other hand, rs2853669, which is in LD with rs2736098 (*r*^*2*^ = 0.79), was shown to be involved in allele specific regulation of telomerase activity in non-small cell lung cancer [65]. Therefore, rs2736098 might be just a tagging SNP of causal variant.

To our knowledge, this is the first such comprehensive study examining association of several potential low-penetrance breast cancer susceptibility *loci* among women in Poland. Beside rs2736098 in *TERT*, only the association of rs3731249 in *CDKN2A* was analyzed previously and significant correlation was identified for early-onset breast cancers [37]. Our study do not confirm this association in any of analyzed models. One of possible explanations of this discrepancy is that more invasive and aggressive types of cancers might have been included in previous study. Also, correction of significance for multiple testing was not conducted in that study, comparing with ours. However, lack of this SNP association, similarly like in case of studied *BRCA1* variants, could be also explained by insufficient study sample size to detect such association (Additional file 2: Table S2) or SNP effect size lower than expected. The rs2736098 in *TERT* locus shows protective effect in both studies and it seems to be specific for the Polish women, indicating the benefit of studying small, homogenous populations for low-penetrance risk variants associations.

## Conclusions

We confirmed the association of four SNPs representing three previously reported susceptibility *loci* with breast cancer risk among Polish women: *FGFR2* (rs1219648 and rs2981582), *TERT* (rs2736098) and 8q24 (rs13281615). Noteworthy is that the minor allele of rs2736098, the synonymous polymorphism in *TERT* gene, was associated with a decreased risk of overall breast cancer, which by now was observed only among women in Poland. Due to the population differences in allele frequencies, identification of general genetic risk factors requires sets of association studies conducted on different populations. Our study confirmed some benefits of studying small and homogenous populations.

## Abbreviations

OR: Odds ratio; SNP: Single-nucleotide polymorphism; GWAS: Genome-wide association studies; CI: Confidence interval; LD: Linkage disequilibrium; ER: Estrogen receptor.

## Competing interests

The authors declare that they have no competing interests.

## Authors’ contributions

Conceived and designed the experiments: JO and EH. Enrolled the patients and performed the experiments: JKL, NM, DN and AN. Analyzed the data: KG, JKL and EH. Wrote the manuscript: JKL and EH. All authors read and approved the final manuscript.

## Pre-publication history

The pre-publication history for this paper can be accessed here:

http://www.biomedcentral.com/1471-2407/13/510/prepub

## Supplementary Material

Additional file 1: Table S1 Primers used for amplification of relevant *BRCA1* and *BRCA2* fragments. Click here for file

Additional file 2: Table S2 The calculation of sample size necessary for given effect detection with study power equal 0.8. Click here for file

Additional file 3: Table S3 SNP associations according to allele frequency test and Cochran-Armitage trend test. Data for all 16 tested SNPs. Click here for file
